# MiR-145 inhibits oral squamous cell carcinoma (OSCC) cell growth by targeting c-Myc and Cdk6

**DOI:** 10.1186/1475-2867-13-51

**Published:** 2013-05-28

**Authors:** Yuan Shao, Yiping Qu, Siwen Dang, Bowen Yao, Meiju Ji

**Affiliations:** 1 Department of Otolaryngology, The First Affiliated Hospital of Xi’an Jiaotong University School of Medicine, Xi’an 710061, the People’s Republic of China; 2 Department of Endocrinology, The First Affiliated Hospital of Xi’an Jiaotong University School of Medicine, Xi’an 710061, the People’s Republic of China; 3 Department of Clinical Medicine, Xi’an Jiaotong University School of Medicine, Xi’an 710061, the People’s Republic of China; 4 Center for Translational Medicine, The First Affiliated Hospital of Xi’an Jiaotong, Xi’an 710061, the People’s Republic of China

**Keywords:** Oral squamous cell carcinoma (OSCC), MiR-145, c-Myc, Cyclin D1, Cdk6

## Abstract

**Background:**

MicroRNAs (miRNAs) are a large group of negative gene regulators that potentially play a critical role in tumorigenesis. Increasing evidences indicate that miR-145 acts a tumor suppressor in numerous human cancers. However, its role in oral carcinogenesis remains poorly defined. The aim of this study is to determine expression levels of miR-145 in oral squamous cell carcinomas (OSCCs) and normal mucosa tissues, and explore its biological functions in OSCCs.

**Methods:**

Reverse transcription quantitative real-time PCR (RT-qPCR) assay was used to evaluate expression levels of miR-145. The biological functions of miR-145 were determined by cell proliferation and colony formation, cell cycle and apoptosis, as well as cell invasion assay.

**Results:**

MiR-145 was frequently down-regulated in OSCCs compared with normal mucosa tissues. Restoring miR-145 expression in OSCC cells dramatically suppressed cell proliferation and colony formation, and induced G1 phase arrest and cell apoptosis. Importantly, our data showed that miR-145 downregulated the expression of c-Myc and Cdk6, which have previously been identified as two direct targets of miR-145.

**Conclusions:**

Our data suggest that miR-145 exerts its tumor suppressor function by targeting c-Myc and Cdk6, leading to the inhibition of OSCC cell growth. MiR-145 rescue may thus be a rational for diagnostic and therapeutic applications in OSCC.

## Background

Oral squamous cell carcinoma (OSCC) is the most common head and neck neoplasm, and the incidence of new cases indicates a continuing rise in developing countries [1]. Although the clinical outcome of OSCC has gradually improved, the overall 5-year survival rate of patients is still disappointing [2,3], reflecting limited advances in our understanding of the pathogenesis of this disease and the molecular events that contributed to its development. Thus, a better understanding of the molecular mechanisms driving oral carcinogenesis may lead to new diagnostic and therapeutic approaches to this disease, and improve the prognosis of OSCC patients.

Like other cancers, oral carcinogenesis involves gradual accumulation of multiple genetic and epigenetic alterations, leading to gain-of-function in oncogenes and loss-of-function in tumor suppressor genes [4,5]. MicroRNAs (miRNAs) are a class of small non-coding RNAs, which play an important role in regulating gene function through targeting mRNAs for translational repression or degradation [6-8]. Abnormalities of miRNA have been implicated in the pathogenesis of a variety of human diseases, notably neoplasms [9-11]. Overexpression of oncogenic miRNAs or underexpression of tumor suppressor miRNAs plays a critical role in tumorigenesis. One major tumor suppressor miRNA, miR-145, which plays a crucial role in regulating smooth muscle cell differentiation [12] and inducing apoptosis [13], is downregulated in many cancers, including prostate, bladder and colon cancer, as well as B-cell malignancies [14-17]. To date, a cohort of genes related to different cancer pathways have been identified and validated as targeted genes of miR-145, such as *Pai-1*, *Fascin1*, *Oct-4*, *Sox-2*, *Klf4*, *c-Myc*, *IRS1*, *Muc1*, *Yes*, *Stat1*, and *p70S6K1*[18-25], suggesting that miR-145 is an oncosuppressor and plays an important role in the initiation and progression of tumor, as supported by several direct evidences that the ectopic expression of miR-145 in cancer cells leads to a loss in cell viability and induces cell death [13,19,26]. It has been well known that downregulation of miR-145 is caused by promoter hypermethylation in several cancers [27,28]. Additionally, the tumor suppressor gene *p53*, which is inactivate in approximately 50% of human cancers, upregulates miR-145 expression, whereas the *Ras* oncogene downregulates its expression [28,29]. Although downregulation of miR-145 is found in an animal OSCC model [30], its role in human oral carcinogenesis remains largely unknown.

In the present study, we investigated expression levels of miR-145 in primary OSCCs and adjacent normal oral tissues using reverse transcription quantitative real-time PCR (RT-qPCR). Our data showed that miR-145 was significantly downregulated in OSCCs compared with normal oral tissues. We also demonstrated the miR-145 suppressed OSCC cell growth by targeting c-Myc and Cdk6.

## Results

### MiR-145 is downregulated in OSCCs

**Figure 1:**
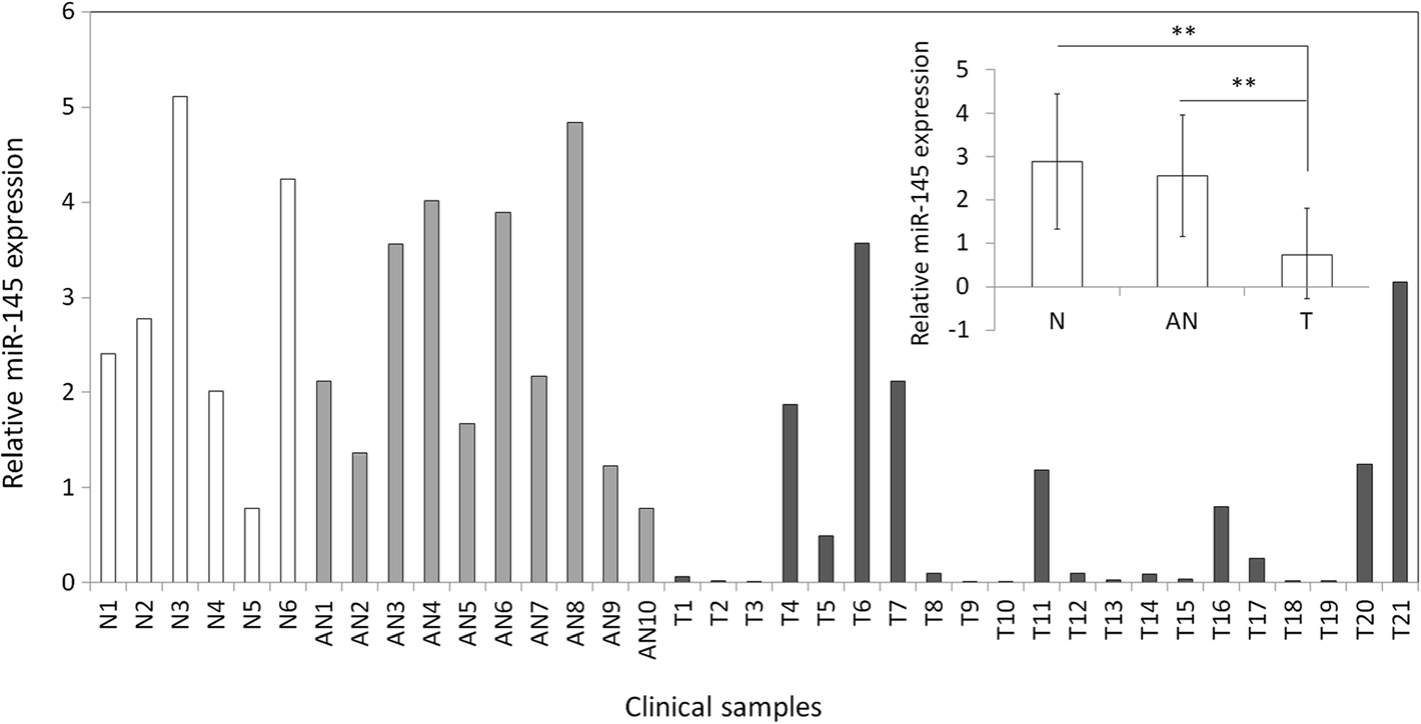
**MiR-145 is downregulated in OSCCs.** Expression level of miR-145 in each individual case of OSCCs, adjacent normal tissues and normal mucosa tissues was evaluated using RT-qPCR. Small nuclear RNA (snRNA) U6 was used as an internal control. Data are presented as mean ± SD (insert). Details are described in the Materials and Methods. T, OSCC tissues; AN, adjacent normal tissues; N, normal mucosa tissues; **, *P* <0.01.

Prompted by numerous studies of miR-145 downregulation in several human cancers [14-17], we sought to identify the role of miR-145 in oral carcinogenesis. We analyzed the expression levels of miR-145 in a cohort of OSCCs, adjacent normal tissues and normal mucosa tissues by RT-qPCR. As shown in Figure 1, miR-145 expression was significantly decreased in OSCCs compared with adjacent normal tissues and normal mucosa tissues. However, there was no significant difference between adjacent normal tissues and normal mucosa tissues. These observations suggest that miR-145 may be an oncosuppressor in this cancer.

### MiR-145 inhibits OSCC cell growth

**Figure 2:**
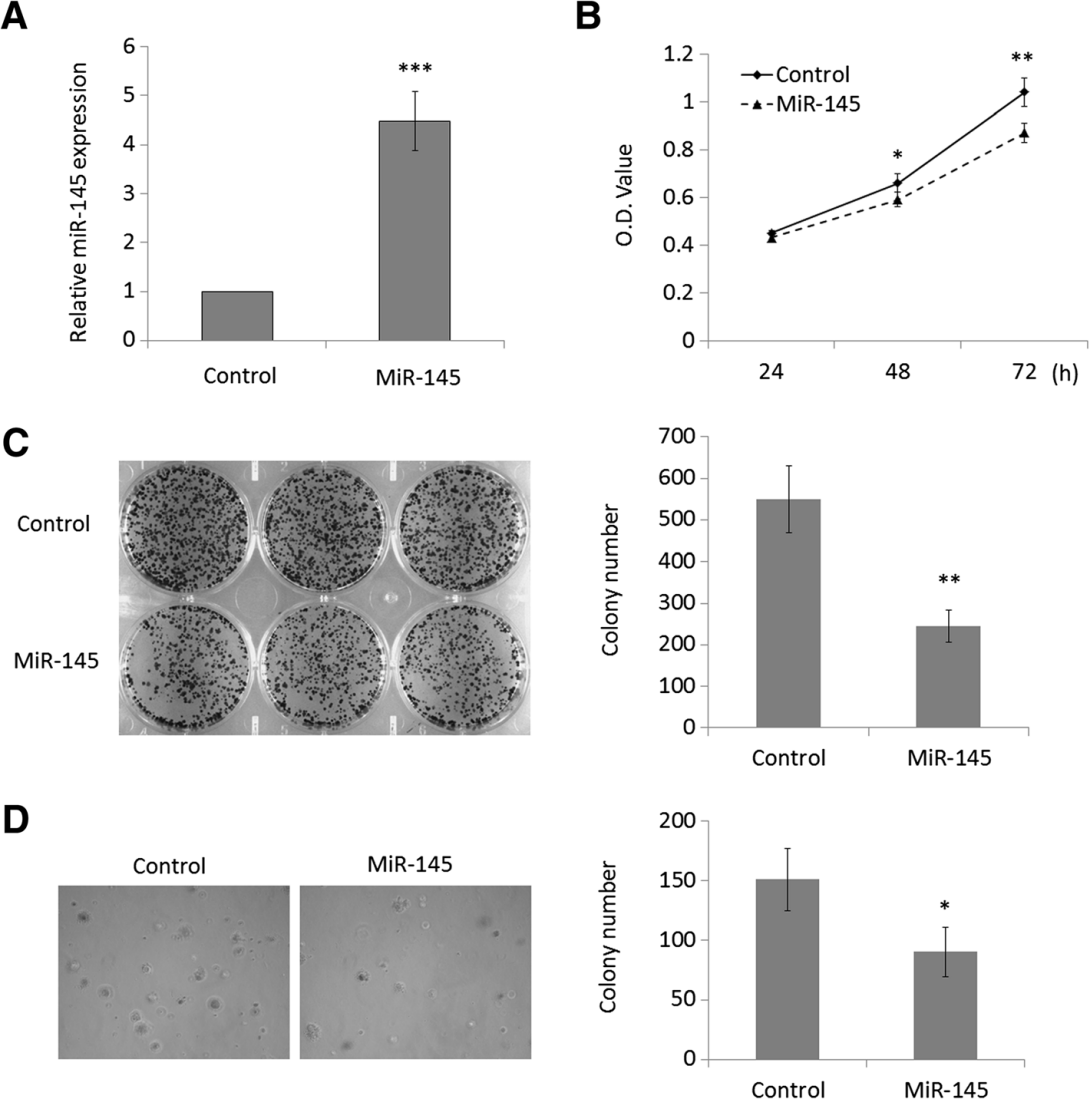
**MiR-145 inhibits OSCC cell growth.** (**A**) Restoration of MiR-145 expression in Tca8113 cells was evidenced by RT-qPCR. SnRNA U6 was used as a normalized control. ***, *P* <0.001. (**B**) MiR-145 significantly inhibited cell proliferation in Tca8113 cells. *, *P* <0.05; **, *P* <0.01. The effect of miR-145 on cell growth was further confirmed by colony formation (**C**) and anchorage independent growth assays (**D**). Left panel showed the representative image of colony formation in Tca8113 cells transfected with miR-145 mimics and miRNA control. Quantitative analysis of colony numbers is shown in the right panel. Details are described in the Materials and Methods. Data are presented as mean ± SD of values from three different assays. *, *P* <0.05; **, *P* <0.01.

To test the effect of miR-145 on OSCC cell growth, we used miR-145 mimics to transfect human OSCC cell line Tca8113. Increased expression of MiR-145 upon transfection was confirmed by RT-qPCR (Figure 2A). As demonstrated by MTT assays, miR-145 restoration dramatically inhibited OSCC cell proliferation (Figure 2B). The inhibitory effect on OSCC cell growth was further confirmed by colony formation and anchorage independent growth assays. Compared to cells transfected with miRNA control, the number of colonies was significantly decreased in cells transfected with miR-145 mimics (Figure 2C and D). Taken together, miR-145 exhibits the growth inhibitory ability in Tca8113 cells and acts as a potential tumor suppressor.

### MiR-145 induces OSCC cell cycle arrest and apoptosis

**Figure 3:**
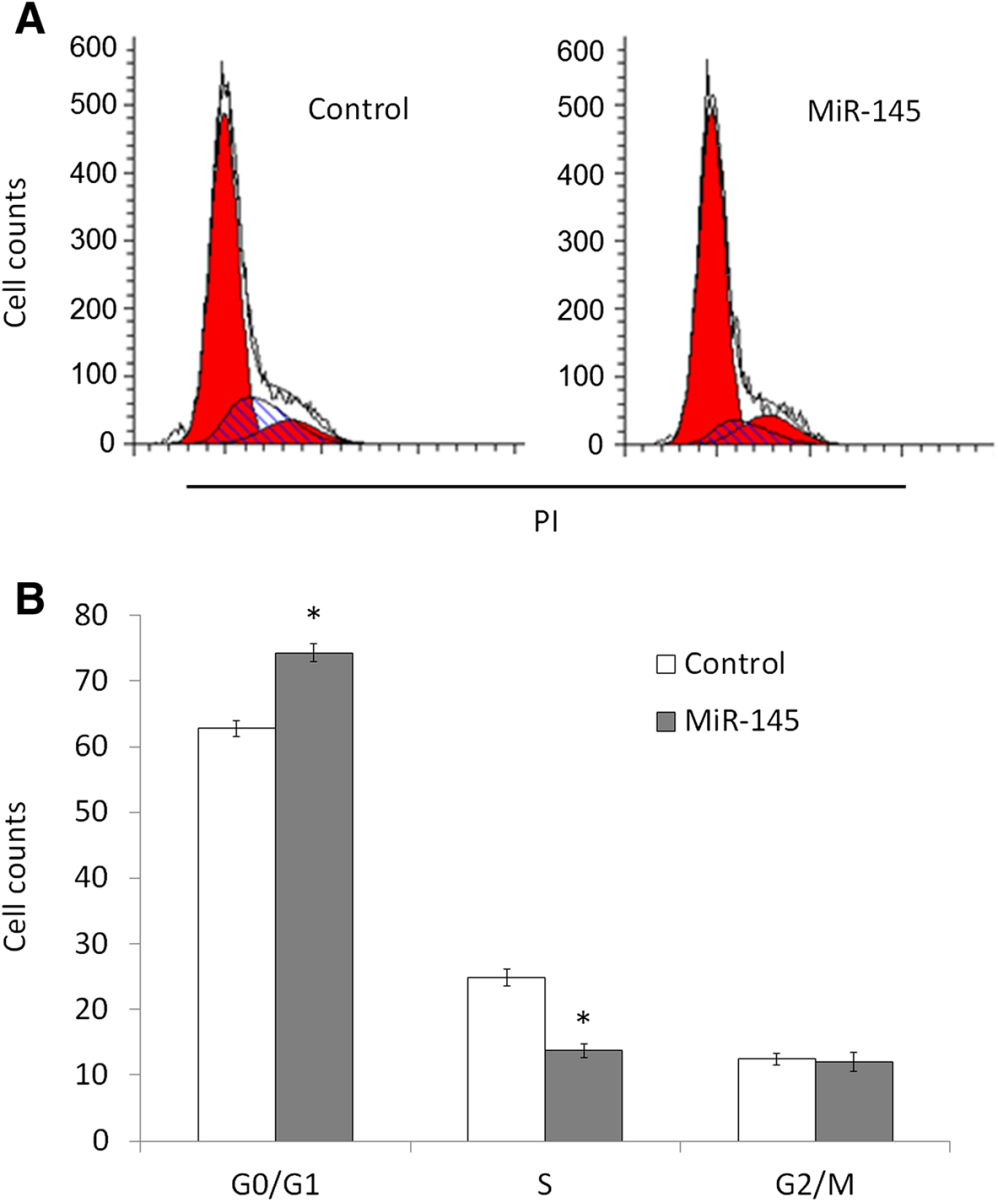
**MiR-145 induces OSCC cell cycle arrest.** Tca8113 cells were transiently transfected with miR-145 mimics and miRNA control. After 72 h post-transfection, DNA content was measured by flow cytometry to determine cell cycle fractions. Representative flow cytometric histograms of cells transfected with miR-145 mimics and miRNA control from three independent experiments are shown in panel **A**. The fraction of cells in each cell cycle phase is indicated in panel **B**. PI, propidium iodide; *, *P* <0.05.

**Figure 4:**
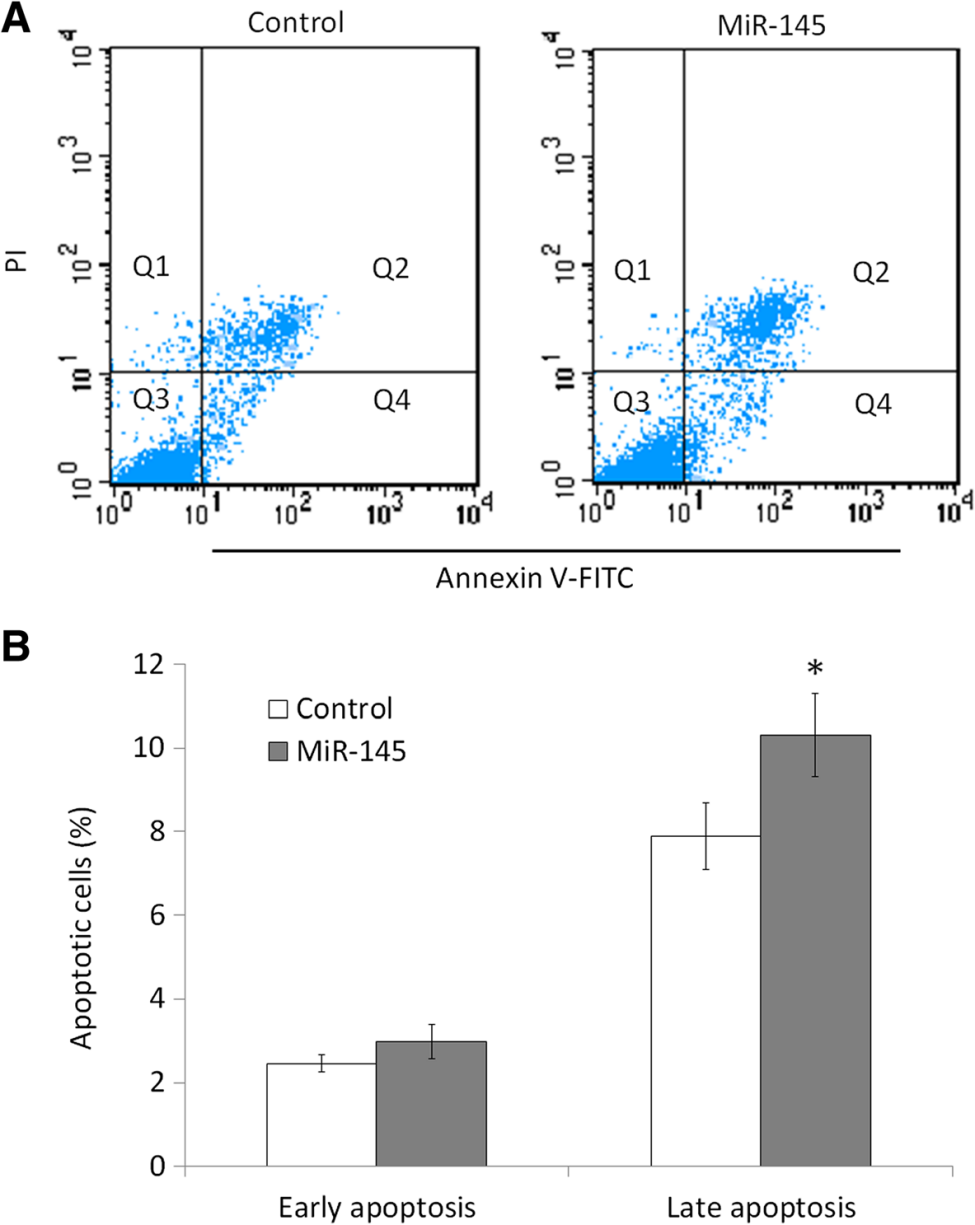
**MiR-145 induces OSCC cell apoptosis.** Cell apoptosis is measured by flow cytometry analysis of Annexin V-FITC double-labeled Tca8113 cells transfected with miR-145 mimics and miRNA control. (**A**) Flow cytometry profile represents Annexin V-FITC staining in *x* axis and propidium iodide (PI) in *y* axis. Dual staining of cells with Annexin V-FITC and PI enabled categorization of cells into four regions. Region Q1 shows the necrotic cells, Q2 shows the late apoptotic cells, Q3 shows the live cells, and Q4 shows the early apoptotic cells. (**B**) The experiment was repeated three times and data represent the average of the early apoptotic and late apoptotic cells. *, *P* <0.05.

Inhibition of cell growth in cancer cells is usually associated with concomitant cell cycle arrest and activation of cell death pathways. We therefore examined the contribution of cell cycle arrest and apoptosis to the observed growth inhibition of miR-145-transfected cells. As shown in Figure 3, compared with miRNA control, cell cycle was arrested at the G1 phase when cells were transfected with miR-145 mimics. The percentage of G1 phase was increased from 62.8% to 74.3%. In addition, the apoptotic cell number increased in miR-145-transfected cells compared with miRNA control-transfected cells, particularly late apoptosis (*P* <0.05) (Figure 4).

### MiR-145 inhibits OSCC cell invasion

**Figure 5:**
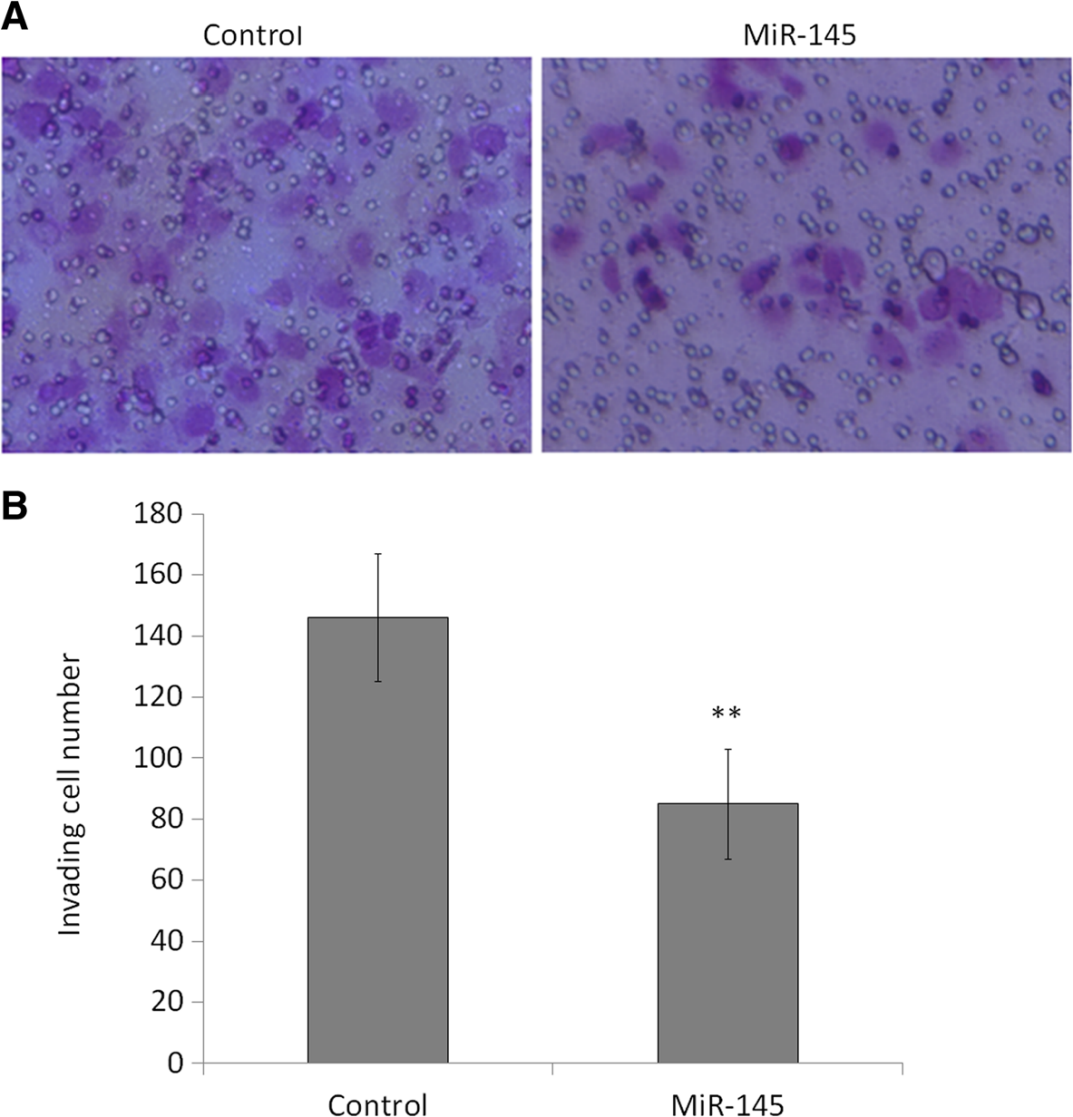
**MiR-145 inhibits OSCC cell invasion.** Cells transfected with miR-145 mimics and miRNA control were starved overnight and then seeded in the Transwell chambers coated with Matrigel for invasion assay. Following a 24 h-culture, non-invading cells in the upper chamber were removed and invading cells were stained and calculated in four microscopic fields per sample. Details are described in the Materials and Methods. Shown are representative images of invading cells (**A**). The bar graphs (**B**), corresponding to upper panels, show means ± SD of the numbers of invading cells from three independent experiments. **, *P* <0.01.

As OSCC is a type of highly malignant tumor with a potent capacity to invade locally and distant metastasis, we next attempted to explore the effect of miR-145 restoration on OSCC cell invasion. As shown in Figure 5, the Matrigel assays showed that the number of cells that passed through Matrigel-coated membrane into the lower chamber was significantly lower in the miR-145-transfected cells than in the miRNA control-transfected cells (*P* <0.001), suggesting that miR-145 inhibited the invasive potential of Tca8113 cells.

### MiR-145 inhibits OSCC cell growth by targeting c-Myc and Cdk6

**Figure 6:**
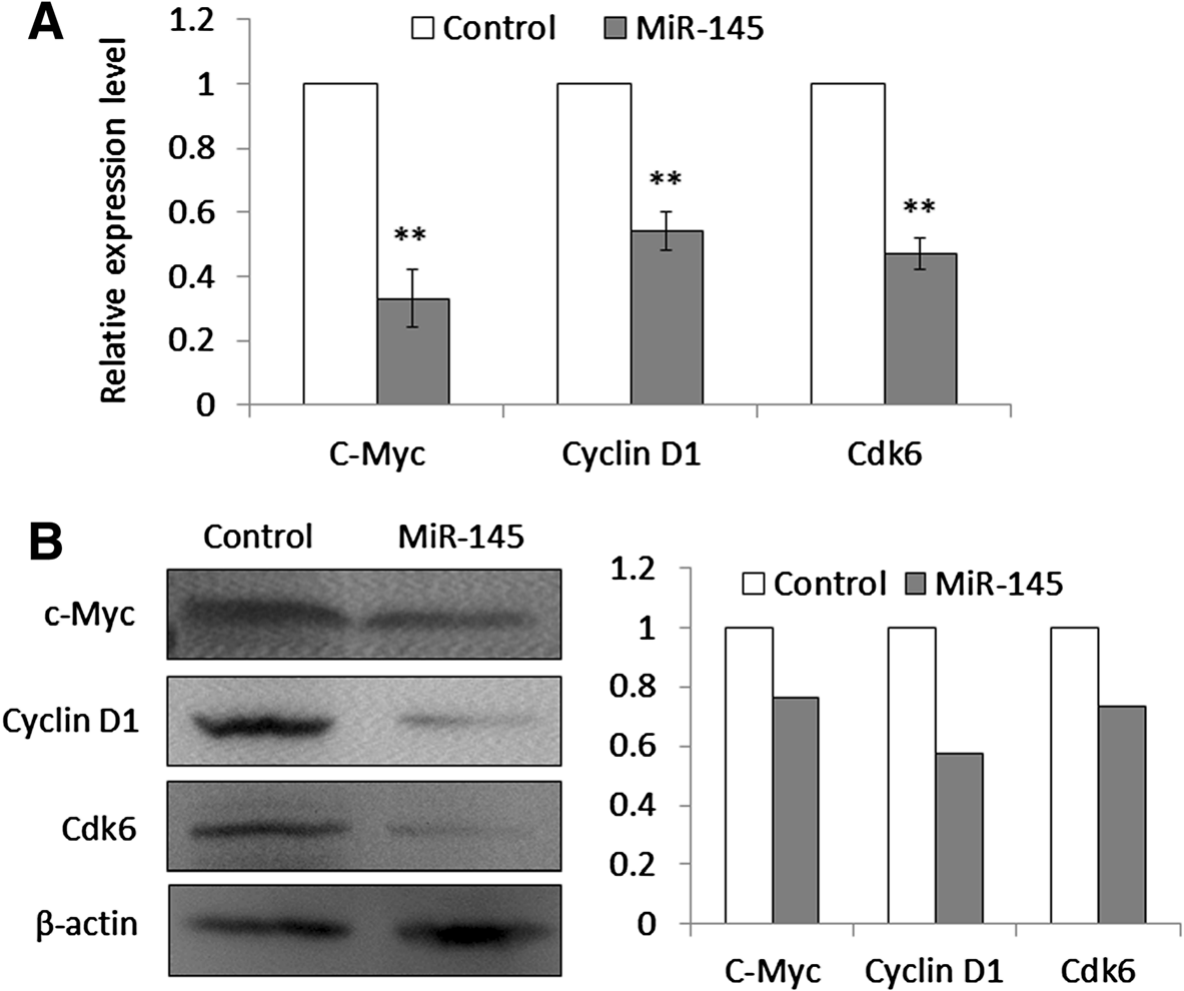
**MiR-145 inhibits the expression of c-Myc, Cyclin D1, and Cdk6 in Tca8113 cells.** Cells transfected with miR-145 mimics and miRNA control were starved overnight and harvested. (**A**) Total RNA was isolated and RT-qPCR was performed to evaluate the expression of c-Myc, Cyclin D1, and Cdk6. Details are described in the Materials and Methods. **, *P* <0.01. (**B**) Cell lysates were collected and subjected to Western blotting assays. The biological functions of miR-145 in Tca8113 cells were determined by blotting c-Myc and its downstream target, cyclin D1, as well as Cdk6. β-actin was used for loading control of Western blotting. Shown in the right portion of the panel is a quantitative illustration of expression of the indicated proteins using densitometry to measure the density of the corresponding bands on Western blot shown in the left portion of the panel.

c-Myc has a pivotal function in growth control, differentiation and apoptosis, and its abnormal expression is associated with many tumors. Thus, we attempt to explore whether miR-145-mediated inhibition of cell growth is due to through targeting c-Myc oncogene. As shown in Figure 6, c-Myc expression was dramatically decreased in miR-145-transfected cells compared with miRNA control-transfected cells whatever the mRNA or protein level. We further confirmed that miR-145 was able to downregulate the target genes of c-Myc, cyclin D1, which were involved in cell cycle regulation. Additionally, miR-145 significantly decreased Cdk6, a major cyclin D-dependent kinase, which was identified as another putative target of miR-145 [31]. These findings suggested that miR-145 suppressed OSCC cell growth, at least in part, by targeting c-Myc and Cdk6.

## Discussion

It has been well established that miRNAs play critical roles in the regulation of cell proliferation, and that miRNA dysregulation is causally involved in the initiation and progression of cancer [9-11]. The interaction networks between miRNAs and transcription regulatory pathways have added a new layer of complexity to regulate cell growth. Recently, changes of miRNA profile in cancer cells and their roles in tumorigenesis have been increasingly appreciated [32,33]. MiR-145 has been reported to be frequently downregulated in various kinds of cancers [14-17]. However, there is presently not very much known about miR-145 involvement in oral carcinogenesis. In the present study, we attempt to investigate miR-145 expression in OSCCs and adjacent normal tissues, and explore its biological function in oral carcinogenesis. Our data show that compared with adjacent normal tissues, miR-145 expression in OSCCs is significantly downregulated, suggesting that miR-145 is a candidate tumor suppressor in the pathogenesis of OSCC.

Down-regulation or silencing of miR-145 may abolish tumor suppression so as to contribute to oral tumorigenesis. We thus test the putative tumor suppressor function of miR-145 in OSCC. MiR-145 restoration in Tca8113 cells shows significant growth-suppressing effect by inhibiting cell proliferation and colony formation in the present study. Furthermore, miR-145 re-expression induces cell cycle arrest and apoptosis, further suggesting its tumor suppressor function. These data are supported by the findings in the other cancers that the ectopic expression of miR-145 in cancer cells leads to inhibition of cell proliferation and induces cell death [13,19,26]. Although the evidences have highlighted the importance of miR-145 as an oncosuppressor in OSCCs, the precise molecular mechanisms remain largely unclear. To better understand the tumor suppressive effect of miR-145 in oral tumorigenesis, particularly inhibition of cell growth, we investigate the effect of miR-145 on c-Myc and Cdk6 in the present study. c-Myc plays its central role in promotion of cell replication by driving quiescent cells into the cell cycle, which is overexpressed in many cancers [34]. This function is originally thought to be elicited mainly via activation of transcription of those c-Myc target genes that are positive regulators of the cell cycle, such as cyclins D1, D2, E and A, *Cdk4*, *e2f1*, *e2f2*, *Cdc25A* and *B*, etc. [34,35]. Cdk6, one of members of the CDK family, is known to play important roles in the cell cycle though binding to cyclin D promotes the phosphorylation of tumor suppressor Rb. Following Rb phosphorylation, cyclin E activates Cdk2 to effect further phosphorylation of Rb, thereby enabling the cells to cross the G1 restriction point [36]. In the present study, our data show that miR-145 downregulates c-Myc and its target gene cyclin D1, as supported by the findings in other cancers that miR-145 represses the expression of c-Myc and its downstream targets in colon cancer and non-small cell lung cancer (NSCLC) [21,37]. Meanwhile, we find that miR-145 dramatically inhibits Cdk6 expression in Tca8113 cells. In line with this study, a previous study has shown that miR-145 inhibits Cdk6 expression by direct targeting its 3’-UTR in colon cancer cells [31]. Taken together, we propose that miR-145 regulates OSCC cell growth, at least partially, by targeting c-Myc and Cdk6, and that loss of miR-145 may provide a selective growth advantage during oral carcinogenesis.

Notably, we find that miR-145 inhibits OSCC cell invasion in the present study. To be consistent with our findings, a previous study shows that miR-145 inhibits the migration of microvascular cells in response to growth factor gradients by directly targeting a transcription factor Fli-1 [38]. Moreover, miR-145-mediated suppression of cell invasion and metastasis is in part caused by directly targeting MUC1 [23]. A recent study also shows that miR-145 affects cell migration of glioblastoma (GB) *in vitro* and *in vivo* by directly targeting NEDD9, implicating an important role of miR-145 in GB invasion [39]. Given the invasive ability is one of the most important features of malignancies, and one of the causes of poor prognosis, thus, miR-145 appears to be a key factor for tumor aggressiveness.

## Conclusions

In summary, our data show that miR-145 is significantly downregulated in OSCCs compared with adjacent normal tissues. To our knowledge, the present of study is the first to demonstrate that miR-145 inhibits OSCC cell growth by targeting c-Myc and Cdk6. As this unique feature of miR-145-mediated gene silencing in human cancers, including OSCC, miR-145 may thus prove to be a potential biomarker for cancer diagnosis and serves as a new target for cancer therapy.

## Materials and methods

### Cell culture and tissues samples

Human OSCC cell line Tca8113 was obtained from China Center for Type Culture Collection (Wuhan, China) and routinely cultured at 37°C in RPMI 1640 medium with 10% fetal bovine serum (FBS). This study was approved by the Institutional Review Board and Human Ethics Committee of the First Affiliated Hospital of Xi’an Jiaotong University School of Medicine. A total of 31 paraffin-embedded tissues, including 21 OSCC and 10 adjacent normal tissues, and 6 normal mucosa tissues were randomly obtained from the First Affiliated Hospital of Xi’an Jiaotong University School of Medicine. None of these patients received chemotherapy and radiotherapy and informed consent was obtained from each patient before the surgery. The histologic diagnosis of tumors was made and agreed upon by at least two senior pathologists at Department of Pathology of the Hospital based on World Health Organization (WHO) criteria.

### Cell transfection

The following siRNA duplexes (GenePharma Inc., Shanghai, China) were used to restore miR-145 expression: MiR-145 mimics, 5’-GUC CAG UUU UCC CAG GAA UCC CU-3’; MiRNA control, 5’-AGG UAG UGU AAU CGC CUU GTT-3’. Transfection of cells was performed using Oligofectamine (Invitrogen, Carlsbad, CA) according to the manufacturer’s protocol. Briefly, cells were seeded in 6-well plates at 30-40% confluence 24 h prior to transfection. MiR-145 mimics and miRNA control (30 nM each) were used for each transfection.

### RNA extraction and reverse transcription quantitative real-time PCR (RT-qPCR)

Total RNA was extracted from cultured cells using TRIzol (Takara Inc., Dalian, P.R. China) according to the protocols supplied by the manufacturers. For paraffin-embedded tissues, total RNA was isolated using the RecoverAll TM Total Nucleic Acid Isolation Kit (Ambion, TX, USA) according to the instructions of manufacturer. The concentration and purity of RNA were determined spectrophotometrically using the NanoDrop ND-1000 (NanoDrop Technologies, DE). cDNA was generated using the PrimeScript® RT reagent Kit (Takara Co., Ltd, Dalian, China) in a 20 μL final reaction volume containing 500 ng of RNA, 0.5 μL PrimeScript® RT Enzyme Mix, and 4 μL 5 × PrimeScript® Buffer, and 1 μL RT primer. Real-time quantitative PCR assay was performed to evaluate miR-145 expression using the SYBR Premix Ex TaqTM II (Takara Co., Ltd, Dalian, China) on an FTC-3000TM System (Funglyn Biotech Inc., Toronto, Canada) according to the instructions of manufacturer. According to the2^-ΔΔCt^ method [40], MiR-145 expression was normalized to small nuclear RNA (snRNA) U6 to calculate the relative amount of RNA present in each sample. The expression values of c-Myc, Cyclin D1 and Cdk6 were normalized to β-actin. Each sample was run in triplicate. The primer sequences were presented in Table 1.

**Table 1:** RT-qPCR primers used in the present study

**Gene**	**Primer sequences (5’-3’)**
MiR-145	RT primer: GTCGTATCCAGTGCGTGTCGTGGAGTCGGCAATTGCACTGGATACGACagggatt
	Forward primer: CAGTGCGTGTCGTGGAGT
	Reverse primer: AGGTCCAGTTTTCCCAGG
U6	RT primer: CGCTTCACGAATTTGCGTGTCAT
	Forward primer: GCTTCGGCAGCACATATACTAAAAT
	Reverse primer: CGCTTCACGAATTTGCGTGTCAT
c-Myc	Forward primer: GCTGCTTAGACGCTGGATTT
	Reverse primer: CACCGAGTCGTAGTCGAGGT
Cyclin D1	Forward primer: AGACCTTCGTTGCCCTCTGT
	Reverse primer: AGTTGTTGGGGCTCCTCAG
Cdk6	Forward primer: TGGAGACCTTCGAGCACC
	Reverse primer: CACTCCAGGCTCTGGAACTT
β-actin	Forward primer: GCACAGAGCCTCGCCTT
	Reverse primer: GTTGTCGACGACGAGCG

### Western blot analysis

Cells were lysed in RIPA buffer. Cellular proteins were collected and subjected to 10% SDS-PAGE, and transferred onto PVDF membranes (Amersham Pharmacia Biotech, Piscataway, NJ). The membranes were then incubated with specific primary antibodies. Anti-c-Myc, anti-cyclin D1 and anti-Cdk6 were purchased from Santa Cruz Biotechnology, Inc. Anti-β-actin was purchased from Epitomics, Inc. This was followed by incubation with horseradish peroxidase-conjugated anti-rabbit or anti-mouse IgG antibodies from Santa Cruz Biotechnology, Inc., and antigen-antibody complexes were visualized using the Western Bright ECL detection system (Advansta, CA).

### Cell proliferation, colony formation and anchorage independent growth assays

MTT assay was performed daily over a 3-d time course to evaluate cell proliferation. Cell culture was added with 10 μL of 5 mg/mL MTT agent (Sigma, Saint Louis, MO) and incubated for 4 h, followed by addition of 150 μL of DMSO and further 15-min incubation. The plates were then read on a microplate reader using a test wavelength of 570 nm and a reference wavelength of 670 nm.

For colony formation assay, cells (5 × 10^5^ cells per well) were seeded in 6-well plates and transfected with miR-145 mimics or miRNA control for 24 h. The medium was refreshed every 3 days. Surviving colonies (≥50 cells per colony) were fixed with methanol, stained with 1.25% crystal violet and counted under a light microscope. For anchorage independent growth assay, 1 × 10^5^ cells with different transfections were cultured into 6-well plates with a bottom layer of 0.6% agar and a top layer of 0.3% agar, respectively. Following the hardening of soft agar, plates were incubated at 37°C with 5% CO_2_. After 2 weeks of culture, colonies were photographed and counted under a light microscope.

### Cell cycle analysis

Seventy-two hours after transfection, cells were harvested, washed twice in PBS, and fixed in 70% ethanol on ice for at least 30 min. Cells were then stained with propidium iodide solution (50 μg/mL propidium iodide, 50 μg/mL RNase A, 0.1% Triton-X, 0.1 mM EDTA). Cell cycles were analyzed based on DNA contents by FACS using a Flow Cytometer (BD Biosciences, NJ).

### Apoptosis assay

Cells were transfected with miR-145 mimics or control for 24 h. Forty-eight hours after transfection, cells were harvested and resuspended with 500 μL of binding buffer. The cell suspension was incubated with 5 μL annexin-V-FITC and propidium iodide at room temperature for 20 min. The stained cells were analyzed on a Flow Cytometer (BD Biosciences, NJ).

### Cell invasion assay

Cell invasion assays were performed using Transwell chambers (8.0 μm pore size; Millipore, MA), which were coated with Matrigel (4 × dilution; 60 μL/well; BD Bioscience, NJ), in 24-well plates. Chambers were pre-coated with rat tail tendon collagen type 1 (0.5 mg/mL) on the lower surface. Transfected cells were starved overnight and then seeded in the upper chamber at a density of 2 × 10^5^cells/mL in 400 μL of medium containing 0.5% FBS. Medium with 10% FBS (600 μL) was added to the lower chamber. Following a 24 h-incubation at 37°C with 5% CO_2_, non-invading cells in the upper chamber were removed with a cotton swab, and invading cells were fixed in 100% methanol and stained with 0.5% crystal violet in 2% ethanol. Photographs were taken randomly for at least four fields of each membrane. The number of invading cells was expressed as the average number of cells per microscopic field over four fields.

### Statistical analysis

All the experiments were similarly done at least three times. Most of the measurements were performed in triplicate and some in duplicates. All statistical analyses were performed using the SPSS statistical package (11.5, Chicago, IL, USA). *P* < 0.05 was considered to be statistically significant. Unless indicated, the results shown in the figures are representatives.

## Competing interests

The authors declare that they have no competing interests.

## Authors’ contributions

MJ conceived and designed the experiments. YS, YQ and SD performed the experiments. MJ and BY collected the samples and analyzed the data. MJ wrote the paper. All authors are in agreement with the content of the manuscript and this submission. All authors read and approved the final manuscript.
